# P-68 - MODEL-BASED EVALUATION OF THE COST EFFECTIVENESS OF TB OUTBREAK MANAGEMENT IN WALES, 2025–2034

**DOI:** 10.1093/pubmed/fdag046.091

**Published:** 2026-07-09

**Authors:** Miss Sophie Carter, Sophie Carter, George Ahern, Dr Matthijs Backx, Dr Daniel Thomas, Wendi Shepherd, Rajendra Kadel, Dr Sean Cavany, Dr Brendan Collins

**Affiliations:** Policy and International Health, a WHO Collaborating Centre on Investment for Health & Wellbeing, Public health Wales; Health Protection Team, Public health Wales; Communicable Disease Surveillance Centre, Public health Wales; Department of Microbiology, Public Health Wales; Communicable Disease Surveillance Centre, Public health Wales; School of Sport and Health Sciences, Cardiff Metropolitan University; Health Protection Team, Public health Wales; Policy and International Health, a WHO Collaborating Centre on Investment for Health & Wellbeing, Public health Wales; Centre for Global Health Research, Nuffield Department of Medicine, University of Oxford; Policy and International Health, a WHO Collaborating Centre on Investment for Health & Wellbeing, Public health Wales; Department of Public Health, Policy & Systems, University of Liverpool

## Abstract

**Background:**

Tuberculosis (TB) incidence in Wales is increasing. To inform future prevention and control strategies, we assessed the cost-effectiveness of TB outbreak investigations.

**Objectives:**

To evaluate the cost-effectiveness of TB outbreak investigations in Wales and evaluate the sensitivity of findings to key parameters.

**Methods:**

Using Welsh TB surveillance data from 2019–2024, we applied a branching process model to simulate outbreaks from 2025–2034. Analyses adopted a health system perspective, with all costs updated using the NHS Cost Inflation Index. We discounted future costs at 3.5% and health outcomes at 1.5%. The primary outcome was the incremental cost-effectiveness ratio. Sensitivity analyses evaluated changes in treatment uptake, transmission, and reductions in infectious period attributable to investigation.

**Results:**

Under the current investigation threshold of ≥2 cases the ICER was £71,300 per QALY gained. Investigating earlier, after ≥1 case, improved cost-effectiveness (£48,485 per QALY), whereas delaying to ≥3 cases reduced cost-effectiveness to £111,133 per QALY. Focusing the ≥2 case threshold on higher-risk populations led to lower ICERs (£51,935 per QALY). Increasing uptake of TB infection treatment from current levels (68–80%) to 90–100% reduced ICERs by at least 15%. Shortening the infectious period further improved cost-effectiveness to £41,339 per QALY. Over a 10-year horizon, projected investigation costs ranged from £1.21M (assuming a 1.5% annual incidence increase) to £4.16M (assuming a 10% increase).

**Conclusions:**

TB outbreak investigations in Wales are resource-intensive, and their cost-effectiveness depends heavily on the investigation threshold. Improved treatment uptake, reductions in infectiousness and earlier investigation substantially improve cost-effectiveness, especially in high-risk groups. These findings will support outbreak management policy-making and underscore the importance of investment in other TB prevention measures, including screening for infection.

